# Supplementary figures and images for: PKN1 Kinase: A Key Player in Adipocyte Differentiation and Glucose Metabolism
Source: Nutrients. 2023 May 22;15(10):2414. doi: 10.3390/nu15102414 (PMC10222094; doi:10.3390/nu15102414)

Supplementary figure S1A

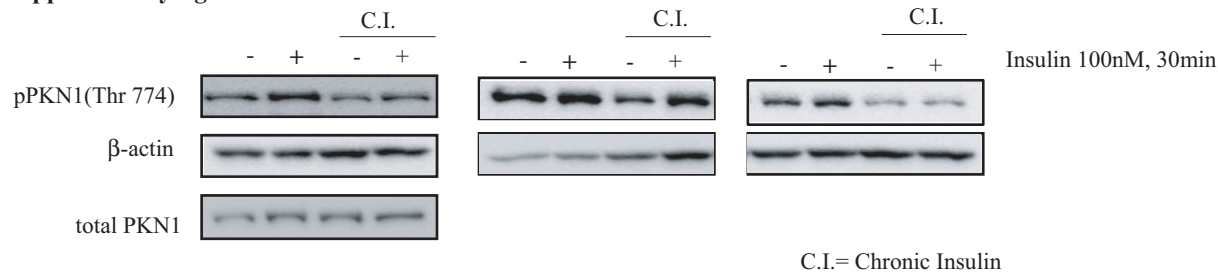

Supplementary figure S1B

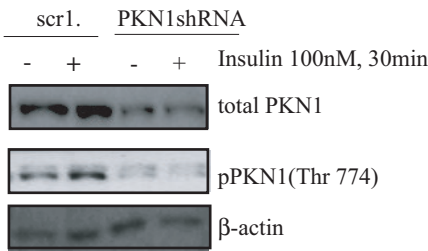

Supplement: Supplementary file 1 [file nutrients-15-02414-s001.zip › nutrients-2389989-supplementary.pdf]
